# A cellular triad for linking cardiac niche to regeneration

**DOI:** 10.1186/s13619-024-00213-x

**Published:** 2024-12-10

**Authors:** Xiaokai Ma, Junjie Hou, Jing-Wei Xiong

**Affiliations:** 1 Beijing Key Laboratory of Cardiometabolic Molecular Medicine, Institute of Molecular Medicine, and College of Future Technology, Peking University, Beijing, 100871 China; 2 School of Basic Medical Sciences, Institute of Biomedical Innovation, The Second Affiliated Hospital, Jiangxi Medical College, Nanchang University, Nanchang, 330031 China

## Abstract

Cardiovascular disease is the leading cause of mortality with very limited therapeutic interventions, thus holding great hope for cardiac regenerative medicine. A recent work from Martin’s laboratory reports their identification of a fetal-like cardiomyocyte progenitor, adult cardiomyocyte type 2 (aCM2), and its potential interactions with C3^+^ cardiac fibroblasts and C3ar1^+^ macrophages to form a regenerative cellular triad, which is only present in the regenerative heart models, YAP5SA-expressing adult hearts and neonatal hearts. The complement signaling is essential for cellular interactions in this regenerative triad. This Highlight summarizes these major findings and provides brief perspectives on the impact of this regenerative niche during cardiac regeneration in the future.

## Main text

Cardiovascular disease is one of the leading causes of death worldwide, featuring myocardial infarction as the most common and devastated disease form (Garbern and Lee 2022). An acute infarction usually results in the loss of a great number of cardiomyocytes. Whereas, adult mammals have limited regeneration capacity to replenish the lost myocardium, leading to permanent scarring and heart failure. Unlike adult mammals, lower vertebrates and neonatal mammals can regenerate their hearts (Porrello et al. 2011; Poss et al. 2002). It is well acknowledged that existing cardiomyocytes in renewable hearts support the recovery of lost myocardium via dedifferentiation and proliferation to generate new cardiomyocytes after injury (Jopling et al. 2010; Kikuchi et al. 2010). Therefore, dissecting the driving force of cardiomyocyte dedifferentiation and proliferation has been actively investigated in the field of cardiac regeneration in the past decades.

Hippo signaling directly regulates cardiomyocyte proliferation in both adult mice and pigs. By phosphorylating its downstream transcriptional coactivator yes-associated protein (YAP), the activation of Hippo signaling instead blocks the nuclear translocation of YAP. Thus, either Hippo suppression or overexpression of YAP5SA, the constitutively activated form of YAP, promotes cardiomyocyte proliferation in adult mice. Activated YAP signaling promotes cardiac regeneration through diverse pathways, such as IGF and Akt signaling (Xin et al. 2011), or by fine-tuning the transcriptomes of genes related to cell-cycle and cytoskeleton organization (Morikawa et al. 2015). Moreover, overexpression of YAP5SA converts adult mouse cardiomyocytes into a more immature and proliferative state, possibly through upregulating the chromatin accessibility at TEAD and AP-1 binding sites (Monroe et al. 2019). In neonatal mice, YAP upregulates cardiomyocyte Wnt signaling to inhibit fibroblast proliferation and cardiac fibrosis (Liu et al. 2021). These works suggest that YAP acts through a domino cascade involving multiple cell types. However, the underlying mechanisms how YAP promotes cardiac regeneration have not been fully elucidated. Recently, Martin and his colleagues report a YAP-induced cardiomyocyte subpopulation, adult cardiomyocyte type 2 (aCM2), and a pro-regenerative cellular triad comprising of aCM2, *C3*^+^ cardiac fibroblast (CF), and *C3ar1*^+^ macrophage (MP) that are only present in the regenerative hearts (Li et al. 2024).

The authors first performed single-cell RNA sequencing and spatial transcriptomics (ST) to map the gene-expression landscapes in two regenerative cardiac models, cardiomyocyte-specific activation of YAP5SA in adult mice and myocardial infarction in neonatal mice. They categorized adult cardiomyocytes into two states, aCM1 and aCM2, in which aCM2 were mostly originated from YAP5SA-overexpressing hearts. Moreover, aCM2 is enriched in fetal structural genes, distinguished from aCM1 which is enriched in oxidative metabolism genes, suggesting that aCM2 is a fetal-like cardiomyocyte population. Immunofluorescence and spatial transcriptomic data showed that aCM2 markers (*Tmsb4x*, *Lmcd1*, and *Acta2*) were weakly located in the subendocardium of control hearts, but were upregulated in YAP5SA hearts. Importantly, they also found that aCM2 signature genes increased in the border zone of human infarcted hearts. The presence of aCM2 in adult mouse and human hearts suggests that fetal-like cardiomyocytes alone are insufficient for regeneration, likely requiring assistance from other cell types.

To address cardiac cellular crosstalks, the authors further explored the datasets from single-cell RNA sequencing and spatial transcriptomics. They found that aCM2-containing ST spots were enriched with the complement pathway genes, which are previously reported to play a role in cardiac regeneration (Natarajan et al. 2018). Among these genes, complement component 3 (*C3*) and its receptor *C3ar1* were specifically elevated in CFs and MPs, respectively, and co-localized with aCM2-containing spots in both adult YAP5SA and neonatal hearts. Furthermore, this cellular triad of aCM2, *C3*^+^ CF, and *C3ar1*^+^ MP was not present in non-regenerative hearts, suggesting a potential role of this niche in promoting cardiac regeneration.

To gain deep insights into the pro-regenerative mechanisms of this cellular triad, they focused on characterizing *C3*^+^ CFs and *C3ar1*^+^ MPs. *C3*^+^ CFs were enriched with a number of anti-fibrotic genes, including *Dcn*, *Igfbp4*, *Cst3*, and *Fstl1*. And *C3ar1*^+^ MPs were enriched with the extracellular matrix and secreted pro-proliferative genes, which have similar transcription profiling to M2 reparative macrophages. Cell–cell communication analysis within this cellular triad revealed multiple interactions that help to promote triad formation (*C3*-*C3ar1* and *Csf1*-*Csf1r*) or to create a pro-proliferative niche (TNF, ADAM, IGF, VEGF, and others). To validate the contribution of *C3*^+^ CFs and *C3ar1*^+^ MPs to cardiac regeneration, by using *C3*^−/−^ and *C3ar1*^−/−^ global knockout mice, they found that deletion of either *C3* or *C3ar1* inhibited YAP5SA-induced MP amplification, cardiomyocyte dedifferentiation and proliferation in adult mice. In neonatal mouse hearts, knockout of either *C3* or *C3ar1* resulted in increased fibrosis and impaired cardiac function recovery after MI. These data strongly support an essential role of the complement system in the formation of pro-regenerative cellular triad and cardiac regeneration.

## Conclusions

Based on two regenerative models (adult YAP5SA and neonatal hearts), combined with multiple technologies including single-cell RNA sequencing, spatial transcriptomics, and loss-of-function mouse experiments, this work reports an interesting fetal-like progenitor aCM2, and its interactions with *C3*^+^ CF and *C3ar1*^+^ MP to form a regenerative triad in the heart. Their data further suggest that the complement signaling is essential for the function of this regenerative triad. While providing meaningful insights into the complement system as an important pathway for YAP to orchestrate CFs and MPs and create a pro-renewal niche, the potential influence of other cell types cannot be ruled out due to the lack of cell-specific knockouts used in this work. Additionally, how YAP activation in cardiomyocytes induced the emergence of anti-fibrotic CFs and anti-inflammatory MPs cannot be fully explained through cell–cell communication analysis alone, warranting further experimental confirmations. Moreover, inflammation and cardiac fibrosis are two important pathological processes following cardiac injury. Like a double-edge sword, how to harmonize these non-myocytes to provide greenhouse for cardiomyocyte proliferation and heart regeneration will be an important direction for future research. Overall, this work proposes an interesting cellular triad for promoting mouse heart regeneration, which are invaluable for the field of heart regeneration in particular and other organ regeneration in general.

## Data Availability

Not applicable.
